# Does blended problem-based learning make Asian medical students active learners?: a prospective comparative study

**DOI:** 10.1186/s12909-019-1575-1

**Published:** 2019-05-15

**Authors:** Ikuo Shimizu, Hideyuki Nakazawa, Yoshihiko Sato, Ineke H. A. P. Wolfhagen, Karen D. Könings

**Affiliations:** 1 Center for Medical Education and Clinical Training, Shinshu University, 3-1-1 Asahi, Matsumoto, 3908621 Japan; 2 Department of Internal Medicine II, Shinshu University, 3-1-1 Asahi, Matsumoto, 3908621 Japan; 3 Department of Internal Medicine IV, Shinshu University, 3-1-1 Asahi, Matsumoto, 3908621 Japan; 4 Department of Educational Development and Research, Faculty of Health, Medicine & Life Sciences, Maastricht University, Universiteitssingel 60, 6229 ER Maastricht, The Netherlands

**Keywords:** Blended learning, Health professions education, Problem-based learning, Quiz, Self-directed learning

## Abstract

**Background:**

Asian educators have struggled to implement problem-based learning (PBL) because students rarely discuss their work actively and are not sufficiently engaged in self-directed learning. Supplementing PBL with additional e-learning, i.e. ‘blended’ PBL (bPBL), could stimulate students’ learning process.

**Methods:**

We investigated the effects of bPBL on tutorial group functioning (discussion, self-efficacy, self-directed learning, active participation, and tutor’s perceived authority) and students’ level of acceptance of the e-learning elements. We compared PBL and bPBL in a medical university in Japan. In the bPBL condition, the tutor’s instructions were replaced with online materials and short quizzes. After the course, a 13-item questionnaire using a 5-point Likert scale was distributed regarding the tutorial group functioning of the tutorial group (influence of discussion, self-efficacy, self-directed learning, active participation, and tutors’ authority). The mean scores of subscales were compared with analysis of covariance. Knowledge levels were measured using a pre-test post-test design. A multiple regression analysis was performed to explore the association between e-learning acceptance and the subscales related to PBL.

**Results:**

Ninety-six students participated in the study (PBL: *n* = 24, bPBL: *n* = 72). Self-efficacy and motivation for learning triggered by group discussions was significantly higher for students in bPBL (*p* = 0.032 and 0.007, respectively). Knowledge gain in test scores was also significantly better in the bPBL condition (*p* = 0.026), and self-directed learning related positively to the acceptance of blended learning (*p* = 0.044).

**Conclusions:**

bPBL seemed more effective in promoting active learning and improving knowledge, without affecting tutors’ authority. Implementing e-learning into PBL is suggested to be an effective strategy in the Asian context.

## Introduction

### Problem-based learning as an example of cultural gap between west and east

Undergraduate medical education in Asia was traditionally characterised by predominantly lecture-based, teacher-centred educational approaches and highly discipline-specific curricula until the late twentieth century, during which the West-inspired theories on learning have been introduced [1]. Problem-based learning (PBL) is one of the typical examples of Western-inspired learning theories. It is defined as the learning that results from the process of working towards the understanding or resolution of a problem [2]. Important goals of PBL include supporting knowledge structuring in clinical contexts, development of clinical reasoning skills and self-directed learning skills, and increasing students’ learning motivation [3]. PBL has been proven equal to lecture-based education in terms of general success [4–8] as well as communication skills, and learning strategies [9–11].

The theoretical origins of the effectiveness of PBL have been variously explored. Among them, self-directed learning is one of key learning principles behind PBL [12]. It is the degree to which students metacognitively, motivationally, and behaviourally participate actively in their own learning process [13]. It refers both to a motivational, volitional component of being willing to engage in learning activities, as well as the ability to do so [14]. Since the amount of knowledge included in standard clinical performance increased rapidly [15], health professions have had to practice life-long learning. Therefore, PBL seems to be a more innovative pathway to learning in medical education, and seemed a convenient and obvious choice for educators to adopt [16]. It has thus been recommended in many countries, including Asia [17]. However, contrary to studies highlighting the success of PBL in Western countries, Asian medical educators have struggled to implement it [18], and Japan is no exception. In Japan, PBL was first incorporated into a curriculum in 1990. It was later suggested that PBL should be implemented in the ‘Model Core Curriculum’, which defined the essential core components of the undergraduate medical curriculum in Japan [19]; PBL was the prevalent educational method at 63 (80%) of the 79 Japanese medical schools in 2004 [20], and 70 in 2016 [21]. However, in 56 of those schools (80%) the implementation of PBL is considered problematic, mainly because of the high burden it places on faculties [22]. As a result, the latest research revealed that a growing number of faculties are avoiding PBL [23], underlining the necessity of coming up with solutions for the implementation problems which have arisen.

Several characteristics of Asian students could be relevant in this matter and should be carefully considered when Asian medical schools are planning and preparing to implement PBL. Students are very deferential towards tutors as authority figures [19]; they fear confrontations with these authority figures and tend to be dependent. There are also vast differences between students’ learning attitudes and their prior knowledge, as well as a lack of passion for what they study during PBL [24]. Effective implementation of PBL builds on students’ prior knowledge and stimulates self-directed learning. Therefore, a lack of prior knowledge could hinder developing one’s own learning objectives in PBL [25]. Conversely, Asian students are so accustomed to the examination-oriented learning culture that they have difficulty applying their prior knowledge and collaborating with peers [26]. In addition, Asian culture predominantly pivots towards teacher-centred pedagogies. In such a prevalent culture, students gain self-efficacy through certified achievements in examinations, rather than through self-directed activities or peer-review, and thus feel less stimulated to develop self-directed learning skills during PBL sessions [27].

### Blended learning

Blended learning is defined as a combination of traditional face-to-face and online instruction [28]. Educational technology offers great flexibility in terms of time and place [29–31] and improves accessibility and opportunities for students to learn regardless of where they are (e.g. campus, hospital, or clinic). The available e-learning materials vary, such as lectures, quizzes, bulletin boards, and online discussion forums. Blended learning has enriched several learning models in various educational strategies, including PBL. Previous studies conducted in the Western context have shown that PBL as a blended learning environment (blended PBL; bPBL) can enhance the satisfaction and self-reliance of participants [32], strengthen students’ commitment [33], and reduce tutors’ presence [34]. The effectiveness of quizzes has been subject to particular scrutiny. A recent meta-analysis revealed that quizzes significantly improve the objective effectiveness and attractiveness of blended learning [35]. Quizzes strengthen learners’ ability to memorise information, which enables easy recall as needed [36]. Feedback on quizzes provides useful information on the correct answers [37]. Additionally, when students are well prepared for face-to-face meetings, these meetings can be used to a larger extent for active learning [38]. Furthermore, task experience can work as performance accomplishment and may be a source of self-efficacy [39]. In terms of the practical relevance of blended learning, better accessibility of online educational materials may enable students to engage in self-directed learning.

While it is important to ascertain whether bPBL is an effective approach for Asian medical students, it is also necessary to understand the drivers which motivate users to accept e-learning in bPBL. Among several theories and models of technology acceptance, the technology acceptance model (TAM) [40] is widely cited in the technology acceptance literature. The TAM explains and predicts user behaviour regarding information technology [41], and provides a basis for tracing how external variables influence perception — particularly perceived usefulness and perceived ease of use — attitude, and intention to use. Some research on e-learning acceptance has been conducted in the context of blended learning [42–44]. For example, learners’ perception of the ease of use of blended learning was associated with self-efficacy [42]. This result is especially relevant for bPBL, as PBL is known to cultivate learners’ attitude towards self-directed learning [3], and learners’ perceived self-directed learning in PBL is positively correlated with perceived self-efficacy [45]. However, learners’ acceptance of e-learning and its effect on bPBL in terms of self-efficacy and self-directed learning as well as discussion activities, active participation, and tutors’ authority have not been examined. Therefore, it is as yet unknown which of these variables is associated with Asian students’ acceptance when e-learning is implemented in PBL. Our study will therefore address the following three research questions:

1. How does bPBL affect students’ perceptions of discussion activities, self-efficacy, self-directed learning, active participation, and tutors’ authority in tutorial group meetings compared to the original PBL?
2. Does bPBL improve Asian students’ knowledge gain compared to the original PBL?
3. Is students’ level of e-learning acceptance of bPBL related to their perceptions of discussion activities, self-efficacy, self-directed learning, active participation, and tutors’ authority?

## Methods

### Context and participants

The context of this study was the PBL programme during the internal medicine clinical rotation of two subspecialties (haematology and endocrinology) for fourth-year students in the six-year undergraduate medical curriculum at a medical school in central Japan. Every student had taken and passed the national summative assessment examinations, called the Common Achievement Tests (CAT), just before starting their clinical clerkship. It was a set of summative assessments comprising a computer-based test (CBT) and an objective structured clinical examination. The tutorial groups were composed of four to six students based on their CBT scores to minimise the difference of average scores within groups.

The PBL programme was conducted in accordance with the widely applied format, referred to as the “seven-jump approach” [46]:

1. Case presentation: The paper-based case is presented and unknown terms are explained.
2. Problem definition: The group defines the fundamental issue.
3. Brainstorming: The students collect ideas.
4. Formation of hypothesis: The results of the brainstorming are formed into a working hypothesis.
5. Defining educational objectives: The students define a detailed agenda to gain a more profound knowledge of the processes forming the crux of the problem.
6. Self-study: The students acquire the necessary knowledge by themselves.
7. Synthesis: The results are presented within the group and the case is revisited based on the insights achieved.

There were two meetings. The first meeting included steps 1–5 and the second meeting included step 7. Between these meetings, students were expected to conduct self-study (i.e. step 6). In the experimental condition, we implemented the bPBL course. The students who were enrolled in the bPBL condition were expected to use e-learning resources in an online environment, which consisted of self-study lecture modules and short quizzes to supplement usual PBL practice. The e-learning environment was based on the institutional learning management system (LMS)—students receive information on the LMS at their freshman orientation and routinely use it in their regular curricula. The tutors were also trained in how to use the system. During the PBL course, three self-study modules were provided. The purpose of the modules was to provide knowledge and improve students’ self-study skills. In order to ensure that students completed the online modules, quizzes related to the learning content were inserted after every few pages in a module. Tutors could check the login information and progress status of students on the LMS. Every module took between 15 to 20 min to complete. Students were expected to spend an average of one hour studying online to prepare for the tutorial meeting. Additionally, the students in the bPBL course could access the online literature resources, which would be in printed versions in the original PBL course.

The students who rotated through departments in October and November 2015 participated in the original PBL condition, and no e-learning module was provided for them. The student groups in the rest of the academic year 2015–2016 participated in the bPBL condition.

Internal medicine specialists participated in the programme as tutors. The same tutors participated in the meetings in both groups. They used the same reference guide for PBL throughout the study [46], and tried to act in a same manner throughout the study. In order to ensure that equal learning opportunities were provided to all students, the blended materials were open to students in the PBL condition afterwards.

**Fig. 1:**
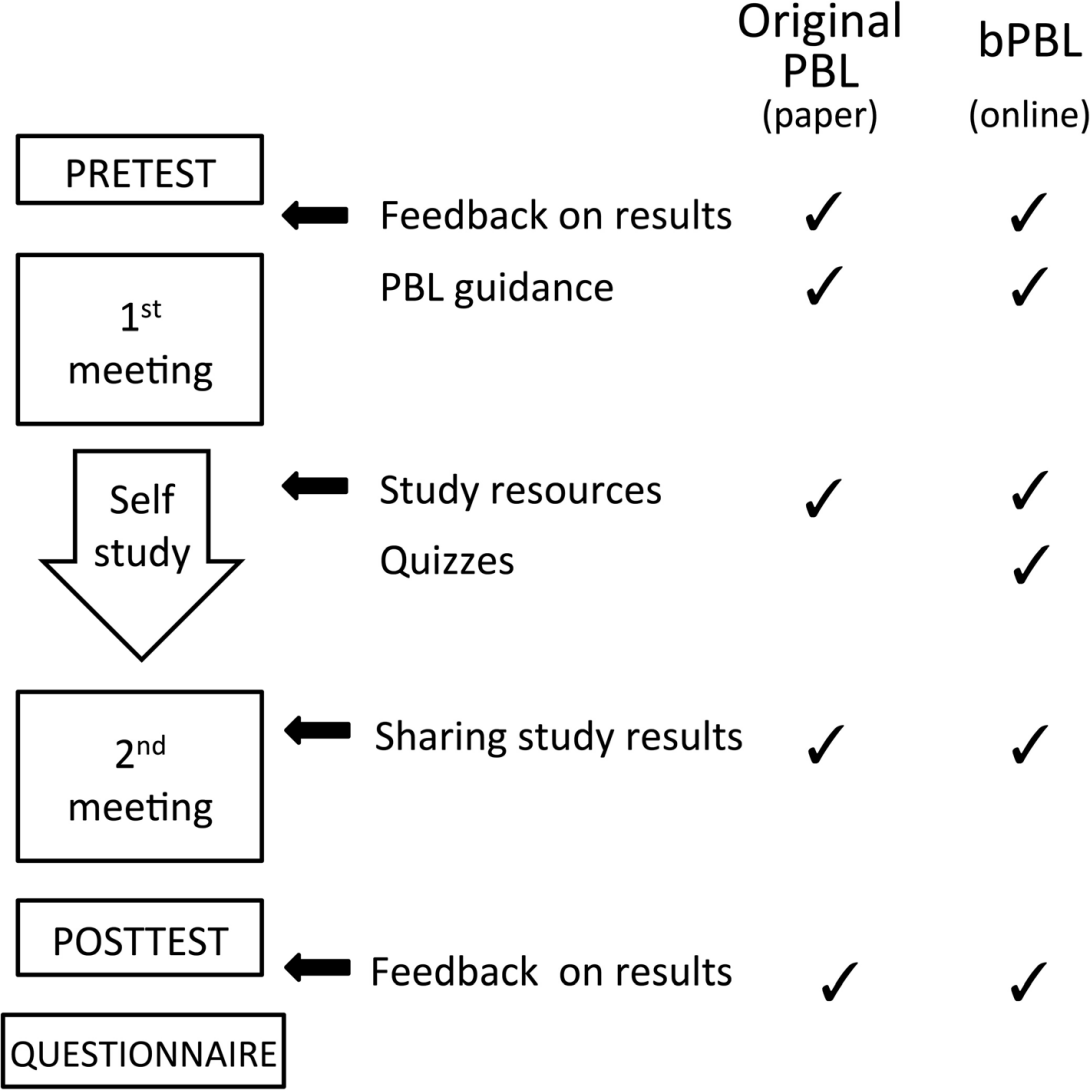
Study context and design. Additional materials were provided by paper (original PBL) or online (bPBL). Online quizzes were provided only in the bPBL condition

Figure 1 provides a summary of the context and study design. In the original PBL condition, students would first take a pre-test, then received feedback and guidance at the first meeting. In the self-study phase, some of the study materials were available from a tutor, while online quizzes were not included. Subsequently, after the self-study phase, students share the knowledge they have acquired with their peers during the second meeting. Finally, the post-test and feedback comments on what they learned and discussed were provided by a tutor. In the bPBL condition, tests, results feedback, and guidance were provided online. The self-study materials were also available online.

### Design and instruments

The research questions were addressed using a quantitative approach. Regarding the first research question, a 13-item questionnaire on tutorial group functioning using a 5-point Likert scale a scale ranging from 1 (*totally disagree*) to 5 (*totally agree*) was administered after each of the two tutorial group meetings. The questionnaire contained six subscales: motivational impact of tutorial group discussions, self-directed learning, self-efficacy during the tutorial, active participation and tutors’ authority [47–50]. As the questionnaire was provided in Japanese, the original English items were translated into Japanese and then back-translated, to confirm the consistency between the original and provided items.

With regard to the second research question, a pre-test and post-test on students’ knowledge level were conducted before the first meeting and after the second meeting, respectively. The purpose of these tests was to measure the students’ knowledge level and evaluate their progress. Questions in the tests were related to the content of the course and consisted of 10 multiple-choice questions adapted from the past national licence examinations. The fundamental recall of prior knowledge was the focus of the pre-test, while higher cognition, including application and analysis of the case problems, were assessed in the post-test. After answering the tests, students received feedback and a short explanation for each question, either face-to-face or online.

For the third research question, four items which represented four constructs in the TAM (ease of use, usefulness, attitude towards the use, and behavioural intention) [40] were included in the questionnaire to identify blended learning acceptance. These items were drawn from a previous study on the acceptance of blended learning [44]. Only students who participated in the bPBL condition were asked to answer these items.

### Analysis

Background characteristics—gender, self-study time, and CAT-CBT—of the two conditions were compared using the chi-square test for gender and the t-test for self-study time and CAT-CBT. The mean scores of each subscale on functioning of the tutorial group (influence of discussion, self-efficacy, self-directed learning, active participation, and tutors’ authority) were compared between the original PBL and the bPBL conditions, by conducting analyses of variance (ANOVA). For the second research question, progress in the test scores (the difference between the pre-test and post-test) were compared between the conditions. Regarding the third research question, a multiple regression analysis was performed to explore the association between e-learning acceptance and the subscales related to PBL (i.e. motivation for learning triggered by group discussions, self-efficacy, self-directed learning, active participation, and tutors’ authority), controlled for gender, subspecialty, self-study time, and prior knowledge). A forced entry method was used for covariates, then a stepwise method was used for the subscales related to PBL. As a measure of effect size, we used eta-squared (*η*^*2*^), where .01 corresponded to a small effect, .06 to a medium effect, and .14 to a large effect [51]. The required sample size was estimated between 52 (with large effect size;*η*^*2*^ = 0.14) and 128 (with medium effect size; *η*^*2*^ = 0.06), when alpha error and power were set as 0.05 and 0.8, respectively. There was no multicollinearity between the measures used. All analyses were performed using SPSS Statistics 23.0.

## Results

Ninety-six students consented to participate in this study (PBL: *n* = 24, bPBL: *n* = 72). Characteristics of the students are shown in Table 1. Students in these conditions did not differ in terms of gender, self-study time during the rotation, or CAT-CBT scores (*p* > .05); thus, the participants in our conditions were similar on these characteristics.

**Table 1:** Students’ characteristics

	Original PBL (*n* = 24)	Blended PBL (*n* = 72)		*df*	*p*
Gender	Male 17	Male 53	χ^2^ = 0.07	1	0.791
	Female 7	Female 19			
CAT-CBT	*M* = 56.654	*M* = 58.039	*t* = − 0.607	33.01	0.548
	*SD* = 10.175	*SD* = 8.003			
Self-study time (hour/week)	*M* = 16.236	*M* = 16.312	*t* = − 0.036	67.58	0.971
	*SD* = 7.295	*SD* = 12.298			

*CAT-CBT* Common Achievement Tests – Computer-Based Testing

The Cronbach’s alphas for the subscales were: 0.843 (motivation for learning triggered by group discussions), 0.724 (self-efficacy), 0.821 (active participation), 0.829 (tutor’s authority), and 0.914 (e-learning acceptance). Thus, their internal consistencies were considered appropriate. Self-directed learning was evaluated with a single item.

### Comparison between original PBL and bPBL

Results on the comparison of the motivation for learning triggered by group discussions, self-directed learning, self-efficacy, active participation, and tutors’ authority are presented in Table 2. Scores on the scales motivation for learning and self-efficacy were significantly higher in the bPBL condition than in original PBL condition (*p* = 0.032 and 0.007, respectively). Also, self-directed learning in the bPBL tended to be higher (*p* = 0.089). There were no significant differences in terms of active participation or tutors’ authority.

**Table 2:** Results of analysis comparing PBL and bPBL conditions

	Mean (SD)		analysis		*η* ^*2*^	power
	PBL	bPBL	*F (1, 90)*	*p*		
Motivation for learning	3.479 (0.961)	3.521 (0.921)	2.412	0.032	0.325	0.830
Self-directed learning	3.049 (0.94)	3.104 (0.897)	2.18	0.089	0.179	0.590
Self-efficacy	3.319 (0.515)	3.597 (0.641)	3.026	0.007	0.405	0.935
Active participation	3.300 (0.64)	3.679 (0.66)	1.306	0.247	0.314	0.654
Tutors’ authority	3.688 (0.998)	3.819 (0.802)	1.089	0.389	0.16	0.406
Progress in test scores	−0.625 (0.998)	0.755 (1.834)	2.277	0.026	0.406	0.893

The progress in test scores was computed as the difference between pre-test (*X =* 5.792, *SD* = 2.245 in original PBL and *X* = 4.994, *SD* = 1.674 in bPBL) and post-test (*X* = 5.167, *SD* = 2.401 in original PBL and *X* = 5.740, *SD* = 1.861 in bPBL). Results of the comparison between the conditions revealed that students in the bPBL condition significantly improved on the test score between the pre-test and post-test (*p* = 0.0026), compared to the PBL condition (see Table 2).

### Association between e-learning acceptance and PBL-related subscales

Level of acceptance of e-learning on average was 3. 120 (SD = 0.912). Multiple regression analysis showed that students’ perception of their self-directed learning related positively to the acceptance of blended learning (*p* = 0.044). No relation between technology acceptance and discussion activities, self-efficacy, active participation, or tutor’s authority was found (Table 3).

**Table 3:** Results of a multiple regression analysis between blended learning acceptance and PBL-related variables (i.e. influence of discussion, self-efficacy, self-directed learning, active participation, and tutor’s authority), controlled for covariates (i.e. gender, prior knowledge, and self-study time)

	*B* (95% CI)		SE *B*	*β*	*p*
Gender	0.093	(−0.417, 0.604)	0.093	0.046	0.716
Prior knowledge (CAT-CBT scores)	0.093	(−0.0417, 0.604)	0.014	−0.445	0.716
Self-study time	−0.011	(−0.045, 0.012)	0.009	−0.445	0.253
Self-directed learning	0.228	(0.006, 0.451)	0.111	0.241	0.044

R^2^ = 0.568

To explore which items of e-learning acceptance were associated with self-directed learning, an additional regression analysis was conducted at item-level. The results (Table 4) revealed that the usefulness of e-learning, in particular, was positively related with self-directed learning (*p* = 0.047).

**Table 4:** Results of a multiple regression analysis between self-directed learning and blended learning acceptance scale items, controlled for covariates (i.e. gender, prior knowledge, and self-study time)

	*B* (95% CI)		*SE B*	*β*	*p*
Usefulness	0.209	(0.003, 0.415)	0.103	0.235	0.047
Ease of use	−0.256	(−0.551, 0.040)	0.148	−0.320	0.656
Attitude towards use	0.287	(−0.177, 0.751)	0.223	0.336	0.537
Behavioral intention to use	−0.110	(−0.526, 0.306)	0.208	−0.128	0.706

R^2^ = 0.470

## Discussion

In this study, we clarified three issues in Asian context; (1) the effect of bPBL on students’ perceptions of discussion activities, self-efficacy, self-directed learning, active participation, and tutors’ authority, (2) students’ knowledge gain in bPBL, and (3) students’ acceptance of bPBL related to their perceptions of discussion activities, self-efficacy, self-directed learning, active participation, and tutors’ authority.

Although several educational methods were developed and implemented successfully in higher education, PBL has proven difficult to implement in Asia. One reason is that an ontological change is necessary for PBL [52], rather than simply introducing its procedures. While the influence of traditional culture such as Confucianism has been assessed [53], similar structural problems have also been observed even in Western cultures [54]. In the present study, instead of intervening in such cultural characteristics, we focused on modifying the PBL programme and attempted to overcome the identified problems such as student participation and engagement by implementing e-learning in the original PBL.

In response to the first research question, which aimed to clarify the effects of adding blended learning elements to PBL on students’ perception of the learning process, our results affirm that it improved self-efficacy and motivational impact of tutorial group discussions. These results may be due to the structural improvement of PBL by implementing e-learning. Instruction and assessment with e-learning complemented supportive information by decreasing the need for tutor-support and providing enactive mastery experience, which was essential for self-efficacy [39] and consequently contribute to self-directed learning.

With regard to the second research question on the effects of bPBL on knowledge improvement, the students using bPBL achieved better progress in test scores, without a difference in study time. A possible reason is that we applied quizzes in the e-learning modules. Evidence of the efficacy in the e-learning quizzes may explain this result [35, 36]. Another reason might be that quizzes were more popular among East Asian students because the examination-oriented learning culture is predominant in most East Asian societies [55], and acts as a school or policy reform barriers in most East Asian societies which rely on examination-centred education [26]. While quizzes are familiar to students, they can also promote the active learning approach [38]. Therefore, if educators try to design blended learning programmes to strengthen active learning for students who prefer examination-centred education, it may be effective to take advantage of the benefits of using quizzes. However, further research is required on how examination-centred educators perceive online quizzes.

Furthermore, as the results relating to the third research question show, self-directed learning is positively associated with blended learning acceptance. We cannot draw conclusions about the direction of this effect. Self-directed students might use bPBL better, but it is also possible that a higher degree of acceptance of bPBL improves students’ self-directed learning.

While previous research suggested that self-efficacy was a strong predictor of perceived ease of use in e-learning acceptance [56], it was not associated with blended learning acceptance in this study. This might be explained by the fact that Japanese students take advantage of extensive prior experience with information and communication technology (ICT) [57]. Park [43] previously reported similar results on e-learning acceptance due to extensive internet experience among university students in Korea, another Asian country with a rich ICT environment. We observed that the usefulness of bPBL was positively correlated with self-directed learning. Usefulness is a fundamental factor in the success and adoption of an e-learning programme [58]. Teachers should commit to purposefully designing e-learning courses to reduce students’ anxiety and to enhance their satisfaction [59].

In contrast to our expectation, we did not observe any significant change in the perception of the authority of the tutor. Because PBL requires knowledge development grounded in discussion and rational argument rather than authority [60], authority has been usually considered a barrier to a suitable PBL environment. Hussain et al. [61] reported that the ‘Asian’ version of PBL requires greater authority on the part of tutors. However, implementing blended learning in PBL does not reduce perceptions of authority. PBL does not abandon all forms of authority: For example, it is the tutor’s role to create a participative, cooperative, reflective, and constructive atmosphere, and there are times when authority is required.

An explanation for our findings is that authority does not always mean authoritarian attitudes [62]. Weber’s model [63] breaks down authority into three types: traditional, legal-rational, and charismatic. Of these three, what happens in the relationship between the teacher and students in a traditional classroom is traditional authority, because the teacher tries to follow his or her management plan based on cultural or learned behaviour [64]. In the traditional learning environment, teachers also rely on charismatic authority to maintain a harmonious environment. These notions are also consistent with the belief that Asian students see teachers as authority figures [50]. On the other hand, legal-rational authority is established on the basis of rational values and established rules. In this type of authority, obedience is impersonal rather than individual [63]. As seen in the observations of a secondary school [64], authority generated from technology is legal-rational, because it functions under a rationally developed rule rather than a person, and can thus be associated with student-centred environments rather than teacher-centred ones. The results of our study, thus, might indicate that blended learning supplements legal-rational authority instead of traditional authority, with possibly remaining charismatic authority for harmony during discussions, and enhances students’ self-directedness through student-centred learning. In the context of bPBL, more empirical evidence is needed to ensure rigour.

Previously, it has been attempted in Asia to improve knowledge-gaining processes in PBL by implementing a hybrid PBL, which is a combined curriculum of lecture and PBL [65]. However, hybrid PBL is still more or less teacher-centred and does not focus primarily on the development of self-directed learning [27]. Our blended PBL with online quizzes was successful in retaining authority, while improving students’ self-efficacy and their learning motivation due to group discussions. As e-learning and online quizzes were provided by the faculty and functioned rationally, discussion grounded by knowledge from such resources might not diminish authority. Our ‘quiz-blended’ PBL is thus suitable for Asian learning environments because it can engage students in PBL while maintaining authority, which is essential in the Asian context.

### Limitations

This study has several limitations. We did a quasi-experimental study and divided groups by rotation schedule. Ideally, it would have been a randomized experimental study. Currently, the possibility of confounding cannot be ruled out. Students differed in their experiences from other rotations and might have communicated with each other about the bPBL, which might have resulted in exchange of additional information. Also, we could not precisely reveal the benefits of blended learning environment because the original PBL did not include quizzes. Furthermore, a benefit of PBL in comparison to traditional learning strategies is its long-term effect [11]. Since we only evaluated after a short period, we recommend future research to investigate long-term effects of bPBL. For drawing stronger conclusions on our research question, a prospective randomized study design should be conducted.

## Conclusions

The results of the study revealed that the effectiveness of PBL can be strengthened by combining with e-learning; moreover, students’ motivation for learning and self-efficacy improved. Thus, the more students are self-directed learners, the higher is their acceptance of the technology in bPBL.

By adding e-learning elements in PBL, we were able to stimulate knowledge-building in a student-centred way, thus supporting self-efficacy and self-directed learning without diminishing tutors’ authority. Therefore, we can conclude that an adjustment strategy of PBL is necessary to adapt it to the Asian context, where greater emphasis has been placed knowledge development grounded in tutors’ authority than in discussion and rational argument. By further improving the design of bPBL to promote the students’ acceptance, this effect could be amplified.

## Abbreviations

ANOVA: Analyses of variance
bPBL: Blended problem-based learning
CAT: Common Achievement Tests
CBT: Computer-based test
ICT: Information and communication technology
LMS: Learning management system
PBL: Problem-based learning
TAM: Technology acceptance model
